# Observations on the Structure of Hydatids

**Published:** 1800

**Authors:** Everard Home


XIX. *Obſervations on the Structure of Hydatids. Extracted from the Croonian Lecture on Muſcular Motion, by* Everard Home, *Eſq. F. R. S. read November* 11, 1790; *and inſerted in the Philoſophical Tranſactions of the Royal Society of London, for the Year* 1795. *Part I.* 4to. London, 1795.

THE ſtructure which produces muſcular action, varies ſo much in different animals, that we are at a loſs to conceive how the effects ſhould have the leaſt ſimilarity; and it is in ſome caſes, only from witneſſing the actions that we can conſider the parts as muſcles: ſince in nothing elſe do they bear a reſemblance to the muſcular ſtructure in the more perfect animals with which we are beſt acquainted.

We ſhall illuſtrate this obſervation by a deſcription of the ſtructure, and actions, of the animals called hydatids, which appear from their ſimplicity to be the furtheſt removed from the human; human; for as the human is the moſt complicated, and moſt perfect in the creation, the hydatid is one of the moſt ſimple, and compoſed of the feweſt parts. It is to appearance a membranous bag, the coats of which are ſo thin as to be ſemitranſparent, and to have no viſible muſcular ſtructure. From the effects produced by the different parts of this bag while the animal is alive, being exactly ſimilar to the contractions and relaxations of the muſcular fibres in the human body, we muſt conclude that this membrane is poſſeſſed of a ſimilar power; and conſequently, has the ſame right to be called muſcular.

The hydatid, from its apparent want of muſcles, and other parts which generally conſtitute an animal, was for a long while denied its place in the animal world, and conſidered as the production of diſeaſe; we are, however, at preſent in poſſeſſion of a ſufficient number of facts, to aſcertain, not only that it is an animal, but that it belongs to a genus of which there are ſeveral different ſpecies.

Hydatids are found to exiſt in the bodies of many quadrupeds, and often in the human; the particular parts moſt favourable to their ſupport appear to be the liver, kidneys, and brain, brain, although they are fometimes detected in other fituations.

One fpecies is globular in its form, the outer furface of the bag fmooth, uniform, and without any external opening; they are feldom found fingle, and are contained in a cyft, or thick membranous covering, in which they appear to lie quite loofe; having no vifible attachment to any part of it. This fpecies is moft frequently found in the liver and kidneys, both of the quadruped and human fubject. They vary in fize, but thofe moft commonly met with, are from one quarter of an inch to three quarters of an inch in diameter.

Another fpecies is of an oval form, with a long procefs, or neck, continued from the fmalleft end of the oval, at the termination of which, by the affiftance of magnifying glaffes, is to be feen a kind of mouth; but whether this is intended merely for the purpofe of attachment, or to receive nourifhment, is not eafily determined. This fpecies is found very commonly in the brain of fheep, and brings on a difeafe called by farmers the ftaggers. It is not peculiar to any one part of the brain, but is found in very different fituations, fometimes in the anterior, at others in the pofterior

 lobe.

lobe. It is inclofed in a membranous cyft like the globular kind; but differs from that fpecies in one only being contained in the fame cyft; and the bag, or body of the animal, being lefs turgid, appearing to be about half filled with a fluid, in which is a fmall quantity of white fediment; while the globular ones are in general quite full and turgid.*

This fpecies, from its containing only a fmall quantity of fluid, has a more extenfive power of action on the bag, and is therefore beft fitted for illuftrating the mufcular power of thefe animals.

If the hydatid be carefully removed from the brain, immediately after the fheep is killed, and put into warm water, it will foon begin to act with the different parts of the body, exhibiting alternate contractions and relaxations. Thefe it performs to a confiderable extent, producing a brifk undulation of the fluid contained in it; the action is often continued for above half an hour, before the animal dies; and is exactly fimilar to the action of mufcles

* The fpecies of hydatid without a neck is alfo met with in the brains of fheep, but is lefs turgid, and lefs of a fpherical figure, than thofe commonly found in the liver.

in the more perfect animals. This ſpecies of hydatid, is very well known by the name *tænia hydatigena*; it varies conſiderably in its ſize; one of thoſe which I examined alive, was above five inches long, and nearly three inches broad at the broadeſt part, which makes it nine inches in the circumference.

The coats of the hydatid, in their recent ſtate, exhibit no appearance of fibres, even when viewed in the microſcope; but when dried, and examined by glaſſes of a high magnifying power, they reſemble paper made upon a wire frame. This very minute ſtructure is not met with in membranes in general; it may therefore be conſidered as the organization upon which their extenſive motions depend.

The coats of the different ſpecies of hydatids had all of them the ſame appearance in the microſcope.

The inteſtines, in ſome of the more delicately conſtructed animals, have a membranous appearance, ſimilar to the bag of the hydatid, and we cannot doubt of their poſſeſſing a muſcular power, ſince there is no other mode of accounting for the food being carried along the canal. The action of the inteſtines, not coming ſo immediately under our obſervation, makes them a leſs obvious illuſtration of this principle than the hydatid; we may, however, conſider their having a ſimilar ſtructure, as a ſtrong confirmation of it.

If we compare the ſtructure of muſcles in the human body, with that of the membranous bag, which compoſes the tænia hydatigena, a ſtructure evidently endowed with a ſimilar principle of action, the theories of muſcular motion, which are founded upon the anatomical ſtructure of a complex muſcle, muſt be overturned.

The ſimplicity of form, in the muſcular ſtructure of this ſpecies of hydatid, makes it evident, that the complex organization of other muſcles, is not eſſential to their contraction and relaxation, but ſuperadded for other purpoſes; which naturally leads us to ſuppoſe, that this power of action, in living animal matter, is more ſimple, and more extenſively diffuſed through the different parts of the body, than has been in general imagined.

